# Metastatic mesenteric dedifferentiated leiomyosarcoma: a case report and a review of literature

**DOI:** 10.1186/s13569-016-0042-6

**Published:** 2016-02-24

**Authors:** Mercy Varghese, Oyvind Bruland, Anne Marit Wiedswang, Ingvild Lobmaier, Bård Røsok, Robert S. Benjamin, Kirsten Sundby Hall

**Affiliations:** Department of Oncology, Oslo University Hospital, The Norwegian Radium Hospital, Ullernchausseen 70, 0379 Oslo, Norway; Institute for Clinical Medicine, Faculty of Medicine, University of Oslo, Box 1171, Blindern, 0450 Oslo, Norway; Department of Radiology, Oslo University Hospital, The Norwegian Radium Hospital, Ullernchausseen 70, 0379 Oslo, Norway; Department of Pathology, Oslo University Hospital, The Norwegian Radium Hospital, Ullernchausseen 70, 0379 Oslo, Norway; Gastrointestinal and pediatric surgery, Section for Hepatopancreatobiliary Surgery, Oslo University Hospital Rikshospitalet, Sognsvannsveien 20, 0424 Oslo, Norway; Department of Sarcoma Medical Oncology, The University of Texas M. D. Anderson Cancer Center, Texas, Houston 77030 USA; Department of Surgery, Nordland Hospital, 8092 Bodoe, Norway

**Keywords:** Leiomyosarcoma, Mesenteric, Abdominal, Histological-subtype, Chemotherapy, Metastases, Multimodal

## Abstract

**Background:**

Abdominal leiomyosarcoma arising from the mesentery is a rare malignancy. It is an aggressive entity with an overall 5 year survival rate between 20 and 30 %. Surgical resection is the cornerstone of primary treatment and may be curative for localized disease. However, patients often develop intra-abdominal relapse and/or metastatic disease. If surgical resection is not feasible, palliative chemotherapy is the treatment of choice. However, there are no clear guidelines regarding chemotherapy; neither in the adjuvant nor advanced setting.

**Case presentation:**

We present a 40 year-old woman, with a mesenteric leiomyosarcoma, who underwent radical tumor resection and did not receive adjuvant oncological therapy. Three months postoperatively, she developed metastatic disease to the lungs and liver. After multidisciplinary assessment she received an unconventional histological-subtype-tailored chemotherapy comprising 3–4 regimens. Initially, there was a decrease both in number and size of metastases. Ultimately, an almost complete radiological response was seen. Subsequent surgical resection and radiofrequency ablation of residual metastatic foci in the liver and lung brought her into complete clinical remission. She is presently tumor free, 36 months following diagnosis of metastatic disease.

**Conclusions:**

To our knowledge, this is the first report of a patient with metastatic mesenteric leiomyosarcoma who is in complete clinical and radiological long-term remission following very aggressive multimodal treatment; including intense poly-drug chemotherapy and without any demonstrable long-term side effects. Given the rarity of mesenteric leiomyosarcoma and lack of guidelines regarding oncological therapy, we suggest that multimodal therapy including aggressive chemotherapy, guided by a multidisciplinary team, is essential to achieve an optimal outcome.

## Background

Leiomyosarcoma (LMS) represents between 10 and 20 % of all newly diagnosed soft tissue sarcomas [1]. It is classified based on anatomical site of origin, a factor that is important for outcome and prognosis [2]. Abdominal LMS is highly aggressive with an overall 5 year survival rate between 20 and 30 % [3]. Although histologically similar, the different anatomic variants of abdominal LMS have varying clinical behaviour and differ in prognosis [4]. Surgical resection is the cornerstone of curative treatment for localized disease if adequate margins are obtained [1, 5]. However, this is often not possible due to anatomical restrictions and large tumor size at diagnosis. Moreover, LMS tends to metastasize, most commonly to the lungs and liver. Unfortunately, there are no clear guidelines regarding the role of oncological treatment; neither in the primary nor metastatic setting [5]. In fact, almost all patients with metastatic disease are considered incurable and, thus, offered only palliative treatment.

Here, we present a patient with an abdominal LMS, specifically a mesenteric LMS. This is a rare malignancy, with only a few cases reported in the literature [6–9]. The LMS was surgically resected with an R0 margin and no adjuvant oncological therapy was given. Unfortunately, the patient developed metastatic disease both to the liver and lungs. Histological evaluation of metastases revealed a highly malignant, pleomorphic sarcoma that resembled her primary tumor, but was not morphologically identical. It was categorized as dedifferentiated LMS [3, 10]. An aggressive multimodal therapeutic strategy was chosen, including staggered intensive chemotherapy tailored to the histological-subtype of the metastasis [11]. Presently, she is in complete clinical remission about 36 months following diagnosis of metastatic disease and without any demonstrable long-term side effects from chemotherapy.

## Case report

Our patient is a 40 year old woman who had no significant medical history except for an appendectomy when she was 20 years-old. She presented with a two-month history of left sided abdominal pain accompanied by abdominal discomfort and distention. Additionally, she suffered from nausea and vomiting without any changes in stool pattern.

**Fig. 1:**
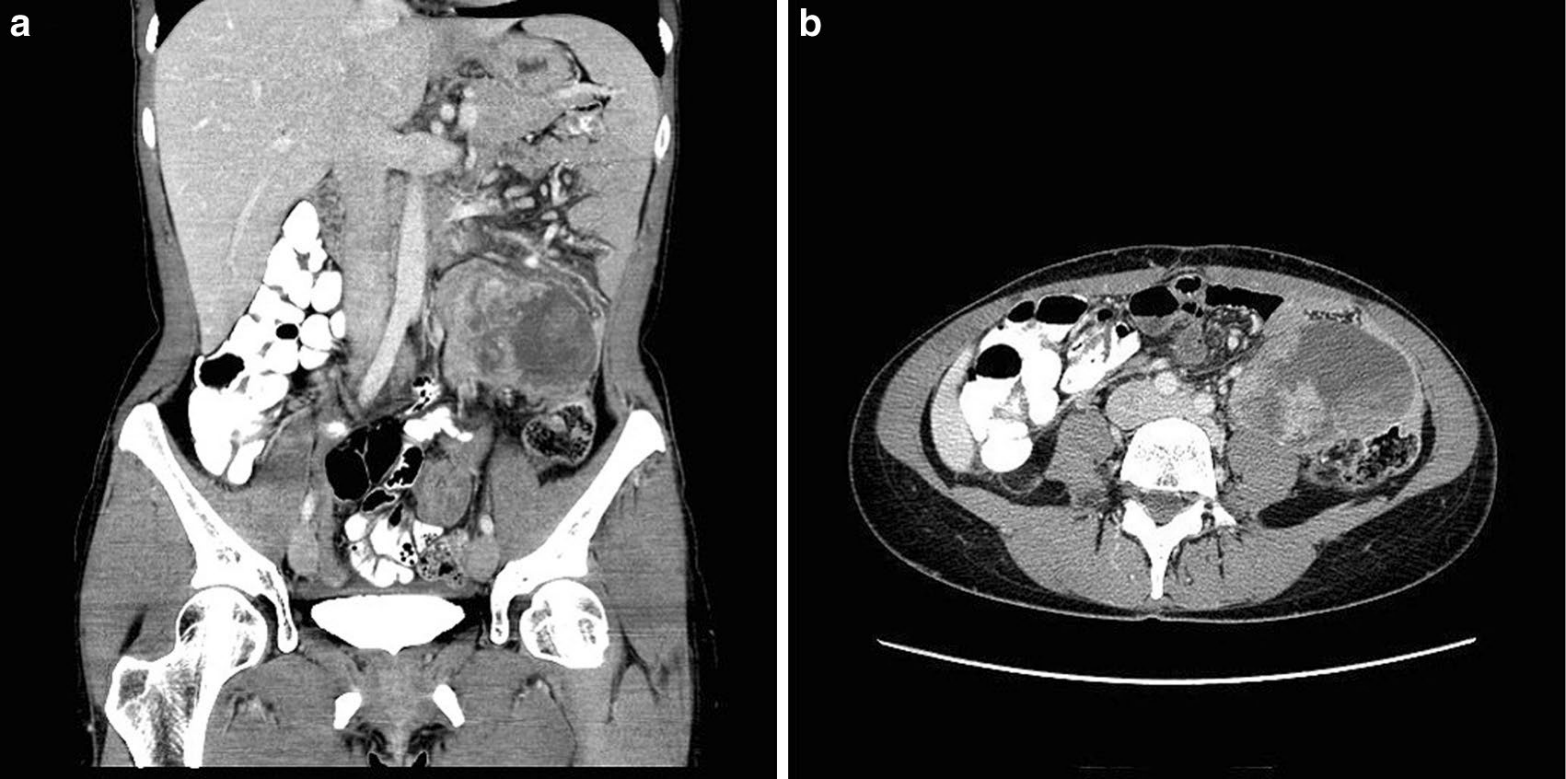
CT of the primary LMS. CT of the abdomen and pelvis showing an intraperitoneal tumor in the left quadrant measuring 8 × 7 × 7 cm (**a** coronal section). The tumor is located in the mesentery and is in close relation to both the small bowel and the sigmoid colon (**b** axial section). The low-density region within the tumor indicates necrosis

On physical examination, a large palpable mass was detected in the left quadrant. This mass was very tender upon palpation, but there were no signs of peritonitis. Blood tests revealed increased acute phase proteins and reduced haemoglobin but were otherwise unremarkable. Computerized tomography (CT) of the abdomen revealed an intraperitoneal tumor in the left quadrant measuring 8 × 7 cm axially and 7 cm craniocaudally (Fig. 1a). The tumor was closely related to the descending colon and small intestine and revealed heterogenous contrast enhancement with necrotic areas (Fig. 1b). There was no pelvic or retroperitoneal adenopathy, though some ascites was seen in the pelvis minor. A sarcoma was suspected and the patient was referred to the Section for Sarcoma at The Norwegian Radium Hospital.

About two weeks later, the patient was operated via a midline laparotomy. The tumor, which was partly adherent to the sigmoid mesocolon, was removed en bloc together with 15 cm of small intestine and colon. An antiperistaltic side-to-side-anastomosis was established at the splenic flexure as well as a small intestinal anastomosis about 120 cm proximal to the ileocaecal valve. No intraperitoneal metastases were seen, but tumor perforation to the peritoneal cavity and resulting bloody ascites was documented perioperatively. Despite this high risk for tumor relapse, no adjuvant therapy was given to the patient in line with guidelines regarding adjuvant therapy for abdominal LMS [5].

**Fig. 2:**
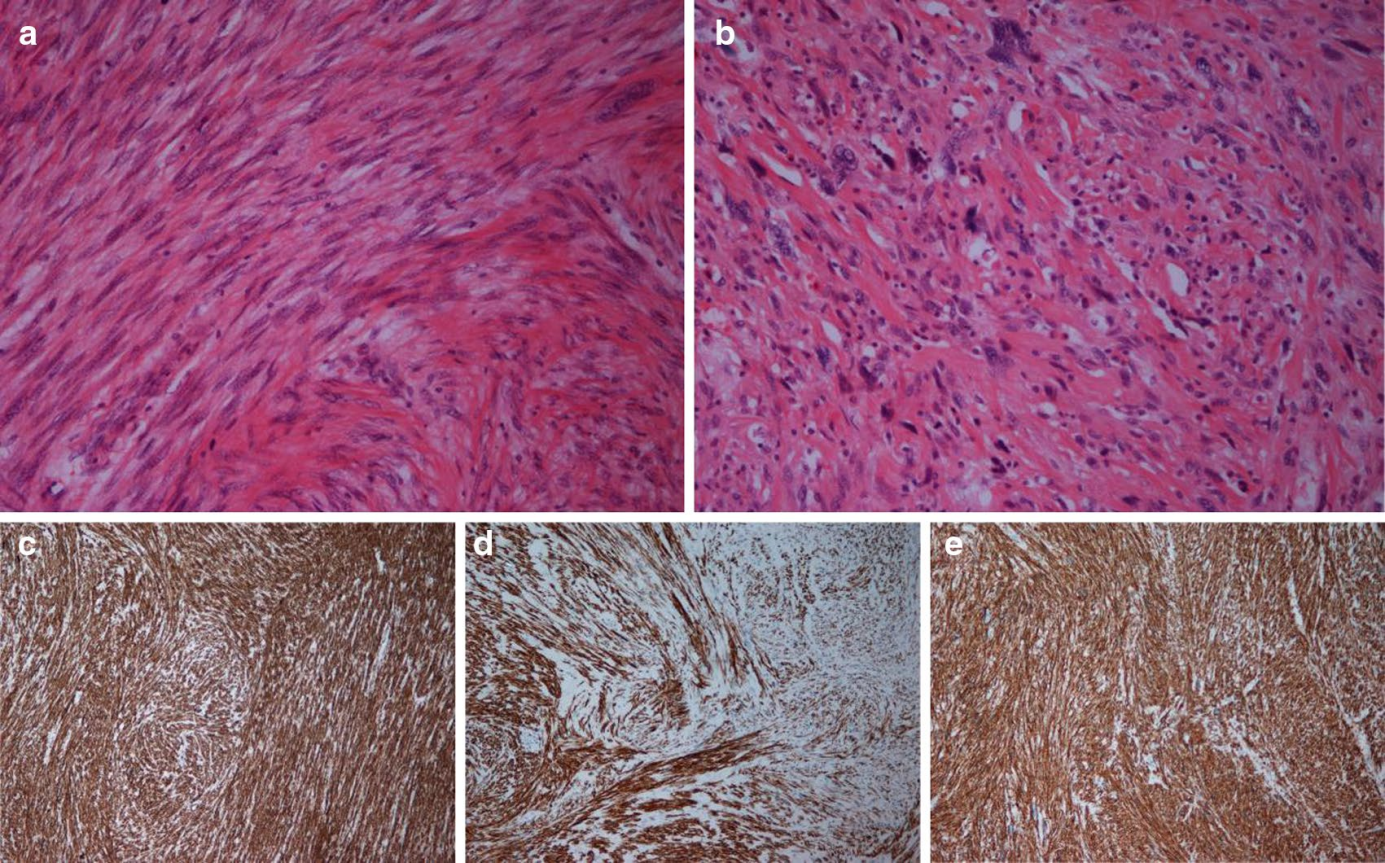
Histopathology of tumor. Histopathological analysis of primary tumor revealed pleomorphic, spindle cells (**a**) and pleomorphic cells (**b**) with eosinophilic cytoplasm consistent with a high grade, pleomorphic leiomyosarcoma. Immunohistochemical examination showed distinct, focal positivity for SMA (**c**), desmin (**d**) and H-caldesmon (**e**), markers that are characteristic for LMS

Histopathological investigations revealed that the resected tumor measured 11 × 9 × 7 cm, was well circumscribed and without infiltration of the small or large intestine. However, the tumor diffusely invaded the serosal surface. More than 50 % of the tumor showed necrosis macroscopically. Histologically, the tumor consisted of pleomorphic, spindle cells with eosinophilic cytoplasm and focally cigar shaped nuclei resembling a high grade, pleomorphic leiomyosarcoma. Up to 14 mitoses per 10 high power fields (1734 mm^2^) were found (French malignancy grade 3) (Fig. 2a, b). No invasion of blood vessels was seen. Immunohistochemical examination showed distinct, focal positivity for caldesmon, SMA and desmin, supporting the diagnosis of LMS (Fig. 2 c–e).

**Fig. 3:**
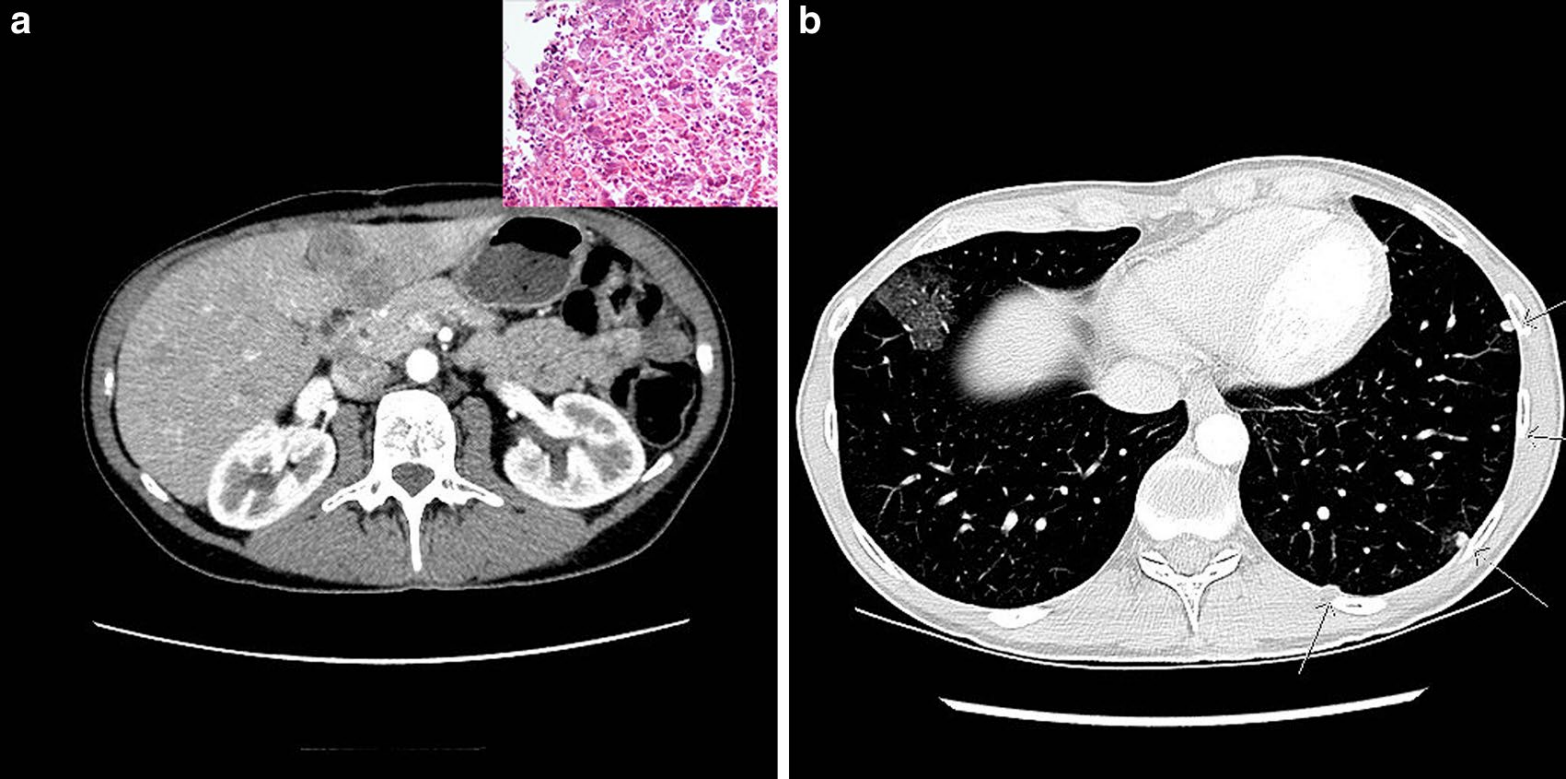
CT of metastatic disease. Axial CT of the abdomen and thorax. Multiple contrast-enhancing lesions in the liver with irregular borders typical for metastases (**a**). Histopathological analysis of a liver metastasis revealed only large, pleomorphic cells (**a** inset) consistent with a highly malignant dedifferentiated pleomorphic sarcoma. CT of thorax showing multiple round, well-circumscribed lung lesions consistent with metastases. The ground-glass opacity around the lesions may be caused by hemorrhage (**b**)

**Fig. 4:**
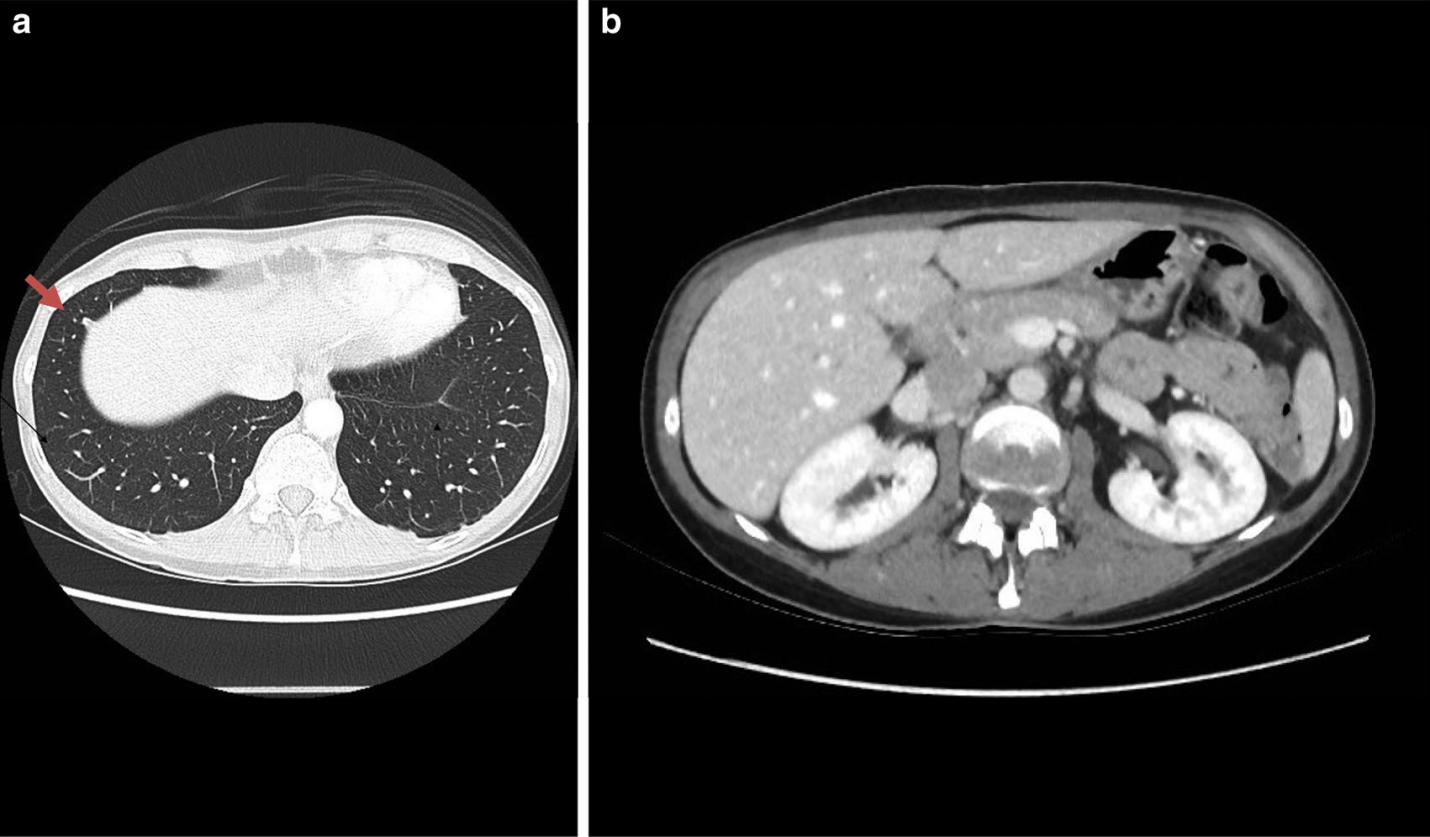
CT showing radiological response. Axial CT of the thorax and abdomen showing almost complete radiological response after histological subtype-specific chemotherapy. A small metastatic lesion measuring 5 × 6 mm (**a**, arrow) and no other visible metastatic foci in the lungs or the liver (**b**) are seen after chemotherapy

About three months after the operation, the patient experienced intermittent abdominal pain. No local tumor recurrence was seen on the CT of the abdomen, but multiple contrast enhancing lesions, typical for metastases, were seen in the liver, the largest measuring close to 3 cm (Fig. 3a). Chest CT revealed multiple round, well-circumscribed lesions in both lungs consistent with metastases (Fig. 3b). Biopsy of a liver lesion confirmed metastatic disease and showed only large, pleomorphic cells (Fig. 3a insert) consistent with a highly malignant pleomorphic sarcoma. After multidisciplinary assessment, we decided to treat the patient with 6 cycles of doxorubicin (50 mg/m^2^, for the first treatment and 75 mg/m^2^ for the other cycles) and ifosfamide (5 g/m^2^) with granulocyte colony-stimulating factor (G-CSF) given at 3 weeks interval. The patient showed a radiological response, exhibiting partial remission with a reduction in size and number of liver and lung metastases (Fig. 4). No new metastases were seen. Moreover, her side effects of treatment were surprisingly modest. Hence, to further enhance the chemotherapeutic effect, we added 5 cycles of weekly doxorubicin monotherapy (20 mg) and 6 cycles with gemcitabine 675 mg/m^2^ days 1 and 8 with docetaxel 75 mg/m^2^ on day 8 and G-CSF on day 9 (every three weeks). This induced a further reduction in the number and size of metastases in the liver and lungs. Due to side effects with water retention and muscle pain we switched to 3 cycles of high dose ifosfamide 12 g/m^2^. Subsequent radiological assessment confirmed an almost complete radiological response to this intense schedule of chemotherapies (Fig. 4).

**Fig. 5:**
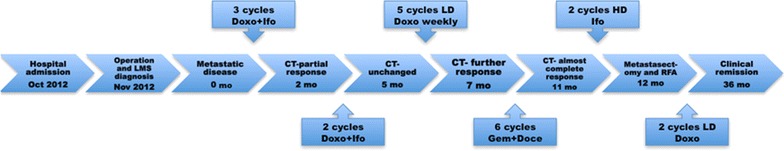
A timeline of events and chemotherapy schedules. Diagnosis of metastatic disease designated as time point zero and denoted as 0 months (0 mo). Milestones within response evaluation and interventional therapy denoted as running months from zero time point. Abbreviations of chemotherapeutic drugs: Doxorubicin and Ifosfamide (Doxo + Ifo), Low-dose Doxorubicin (LD Doxo), Gemcitabine and Docetaxel (Gem + Doce), High-dose Ifosfamide (HD Ifo)

After a multidisciplinary discussion, we considered the remaining visible lesions in the lungs and liver to be resectable. Therefore, the patient underwent combined left hepatectomy in combination with radiofrequency ablation of metastatic foci in the right liver lobe, followed by a thoracoscopic wedge resection of metastasis in the right lung. The rationale of removing metastases was to decrease tumor burden, as this has been shown to prolong disease-free survival and probably also overall survival, similar to what is seen in patients who undergo resection of metastases limited to the lungs [12]. This combination of surgery and/or radiofrequency ablation of residual lesions was performed to improve chances of long-term clinical remission, as there was no evidence of tumor outside the liver and lungs. An immediate postoperative MRI of the abdomen showed three residual lesions in the liver which were not visible on the perioperative ultrasound and suspicious subcutaneous lesions near the midline laparotomy scar. A repeat laparotomy was then performed in which the abdominal scar was removed in combination with ultrasound/MRI-image fusion guided re-ablation of the liver. Histological evaluation of the aforementioned lesions revealed only fibrotic changes with no viable tumor cells. Postoperatively, the patient received 2 cycles of weekly doxorubicin 20 mg due to a strong desire from the patient. Post operative CT of the abdomen and thorax revealed only postoperative changes in the liver and lungs. A complete outline and scheduling of the various chemotherapies are displayed in Fig. 5.

Five months after surgery for metastatic disease, the patient developed abdominal pain and constipation. CT of the abdomen and thorax revealed only postoperative changes. Moreover, upper and lower endoscopy was unremarkable. A contrast study showed no pathology, only stenosis of the small intestine, which was dilatated, and the patient showed symptomatic relief after laxative therapy. The patient is presently in clinical remission, 36  months following diagnosis of metastatic disease.

## Discussion

### Abdominal LMS

Mesenteric LMS was first described in 1963 [13] and is most likely derived from the smooth muscle cells of blood vessels in the mesentery [3]. It is an aggressive disease and approximately half of all patients will develop distant metastases despite adequate local control [14]. The overall 5 year survival rate is only 20–30 % [3], partly because mesenteric LMS often remains undetected until late in the course of disease development, due to the large available space of the abdominal cavity. As in the case of our patient, abdominal distension and discomfort are common presenting symptoms [1]. Additionally, altered bowel movements and weight loss are also seen. Blood tests are non-specific but may reveal anaemia [1] and elevated acute phase proteins. Radiological assessment is mandatory for preoperative evaluation of tumor location, dimensions and relation to adjacent structures. Definitive diagnosis is based on histopathology.

### Treatment of abdominal LMS

#### Localized disease

Surgery is the cornerstone of treatment and is the only therapeutic modality proven to provide cure [5]. However, complete resection is often difficult due to large tumor size at presentation and recent studies have shown that resection margins may be the only significant predictor of local recurrence [4, 15]. Moreover, complete primary surgical resection is critical for achieving the best overall outcome [16]. Other factors include tumor depth, histological grade, and metastasis at presentation [15, 17]. The role of adjuvant chemotherapy in localized disease is not established and therefore is not standard treatment [5]. It can be proposed to high-risk patients, after multidisciplinary assessment or within clinical trials. In keeping with current guidelines, our patient did not receive adjuvant treatment.

### Metastatic disease

#### Chemotherapy

When our patient developed metastatic disease, a core needle biopsy of a liver metastasis was obtained. Though the biopsy material was limited, a highly malignant tumor, displaying only large pleomorphic cells similar to the cells focally present in the primary tumor, was seen. This may suggest a histological-subtype that had evolved into a dedifferentiated phenotype compared to the primary tumor. This was the reason for choosing to add ifosfamide, rather than dacarbazine, to the initial regimen [18]. Such biological heterogeneity is documented between primary and metastatic disease in a number of malignancies [19]. Patients with pleomorphic or dedifferentiated LMS have a particularly poor prognosis, with a metastatic rate of 89 % [10].

The choice of chemotherapeutic agents depend on several factors including histological-subtype, age, co-morbidity and expected tolerance of side effects. Our present knowledge regarding chemotherapy of LMS is mainly based on non-randomized phase II studies or retrospective case series [11]. Furthermore, the rarity and heterogeneity of soft tissue sarcomas, as well as the high variability of patient characteristics, chemotherapeutic regimens, histological and molecular subtypes (early trials might even have included GIST patients), the variable follow-up and differing definitions of end points all preclude the available evidence of chemotherapeutic effectiveness.

Despite the paucity of randomized trials, anthracyclines, either as a single-or-multi-agent chemotherapy, are the first-line of treatment for abdominal LMS [5], according to the ESMO-guidelines. Moreover, depending on the histological-subtype, doxorubicin and dacarbazine (for a purely LMS) [20] or doxorubicin and ifosfamide (for malignancies with a pleomorphic cellular component) [21] have been advocated. Second-line chemotherapeutic agents recommended include (1) standard ifosfamide or high-dose ifosfamide (around 14 g/m^2^) [22], (2) a combination of gemcitabine and docetaxel [23] (which showed improved progression free survival and overall survival than gemcitabine alone) [24], (3) a combination of dacarbazine and gemcitabine [25] and (4) trabectedin [26]. The rationale for giving the patient such intense and sequential chemotherapy has previously been shown to be successful [27] in achieving a maximal radiological and clinical response. It has also been reported to improve long-term survival [28]. We chose not to use trabectedin and pazopanib as there is not much experience with these drugs in a curative treatment setting.

For advanced metastatic disease, surgery or radiofrequency ablation is usually not the treatment of choice, but may be considered in selected cases where the number of metastatic foci are few and amenable to intervention. Given the remarkable response of our patient to chemotherapy, we decided to remove remaining lesions in the lung and liver using surgical resection and radiofrequency ablation with the hope of achieving long-term, complete clinical remission [29]. The rational for such an approach is that the smallest lesions, including micrometastases, may have been completely eradicated by the chemotherapy. Hence, removing the larger metastases, which may still contain foci of viable sarcoma cells, eliminates the nidus for subsequent relapse. While the degree of response to preoperative chemotherapy has not been demonstrated to influence post-resection survival, largely due to small sample size, the progression of disease prior to resection is a known adverse prognostic factor [30]. The presence of disease outside the chest has been considered a contraindication to resection of pulmonary metastases. More recently, however, it has been shown that resection of pulmonary and extrapulmonary metastases results in disease-free survival and overall survival, similar to that seen in patients who undergo resection of metastases limited to the lungs [12].

## Conclusions

To our knowledge, this is the first report of a patient with metastatic mesenteric LMS who developed complete clinical and radiological remission without any demonstrable long-term side effects from chemotherapy, after receiving multimodal therapy including an unconventional histological-subtype-tailored chemotherapy comprising 3–4 regimens, surgical resection and radiofrequency ablation of metastatic foci. Given the rarity of mesenteric leiomyosarcoma and lack of guidelines regarding oncological therapy, we suggest that multimodal therapy including aggressive histological-subtype tailored chemotherapy can result in complete remission under the guidance of a multidisciplinary team.
